# A systematic review and meta-analysis of hernia sac management in laparoscopic groin hernia mesh repair: reduction or transection?

**DOI:** 10.1186/s12893-023-02147-8

**Published:** 2023-08-23

**Authors:** Mohamed Ali Chaouch, Mohammed Iqbal Hussain, Amine Gouader, Abdallah Amine Lahdhiri, Alessandro Mazzotta, Adriano Carneiro da Costa, Bassem Krimi, Faouzi Noomen, Hani Oweira

**Affiliations:** 1 Department of Visceral and Digestive Surgery, Fattouma Bourguiba Hospital, University of Monastir, Monastir, Tunisia; 2 Department of General Surgery, Portsmouth Hospitals University NHS Trust, Portsmouth, UK; 3 Department of Surgery, Perpignan Hospital Center, Perpignan, France; 4 Department of Anesthesia and Intensive Care, Farhat Hached Hospital, University of Sousse, Sousse, Tunisia; 5 Department of Digestive, Metabolic, and Oncologic Surgery, Institute Mutualist of Montsouris, Paris, France; 6 Department of Surgery, Universitäts medizin Mannheim, Heidelberg University, Mannheim, Germany

**Keywords:** Hernia repair, Reduction, Transection, Total extraperitoneal, Transabdominal preperitoneal, Seroma, Outcomes

## Abstract

**Background:**

There is no consensus regarding hernia sac management during laparoscopic hernia repair, and this systematic review and meta-analysis aimed to compare the postoperative outcomes of sac reduction (RS) and sac transection (TS) during laparoscopic mesh hernia repair.

**Methods:**

We conducted a systematic review and meta-analysis according to the Preferred Reporting Items for Systematic Review and Meta-analysis (PRISMA) 2020 and AMSTAR 2 (Assessing the Methodological Quality of Systematic Reviews) guidelines. We used the RevMan 5.4 statistical package from the Cochrane collaboration for meta-analysis. A random effects model was used.

**Results:**

The literature search yielded six eligible studies including 2941 patients: 821 patients in the TS group and 2120 patients in the RS group. In the pooled analysis, the TS group was associated with a lower incidence of seroma (OR = 1.71; 95% CI [1.22, 2.39], p = 0.002) and shorter hospital stay (MD = -0.07; 95% CI [-0.12, -0.02], p = 0.008). There was no significant difference between the two groups in terms of morbidity (OR = 0.87; 95% CI [0.34, 2.19], p = 0.76), operative time (MD = -4.39; 95% CI [-13.62, 4.84], p = 0.35), recurrence (OR = 2.70; 95% CI [0.50, 14.50], p = 0.25), and Postoperative pain.

**Conclusions:**

This meta-analysis showed that hernia sac transection is associated with a lower seroma rate and shorter hospital stay with similar morbidity, operative time, recurrence, and postoperative pain compared to the reduction of the hernia sac.

**Protocol:**

The protocol was registered in PROSPERO with ID CRD42023391730.

## Introduction

Groin hernia repair is one of the most commonly performed surgical procedures. However, the optimal surgical procedure remains controversial [1]. Moreover, each procedure includes some technical variations: surgical approach [2], mesh types [3], mesh fixation modalities [4], mesh no fixation [5], and attitude regarding the hernia sac [6]. These varieties were developed to reduce postoperative complications, pain, recurrence, return the patient to normal activities quickly, improve quality of life, and minimize postsurgical discomfort as well as the adverse effects of surgery. It is widely accepted that the laparoscopic approach is safe, reproducible, and associated with enhanced recovery and less postoperative pain [1]. However, many studies have highlighted the limitation of a longer operative time, particularly due to hernia sac manipulation and reduction [7]. This dissection is performed in a larger preperitoneal plan than in the open approach, which requires the dissection of the hernia sac from the spermatic cord without separating the preperitoneal space. It is sometimes difficult to achieve total reduction with sac transection and distal splitting, especially in large indirect inguinal sacs and inguinoscrotal sacs. However, prolonged and extensive laparoscopic dissection of the herniated sac increases the risk of damage to the testicular vascular supply or the vas. We postulated that laparoscopic sac transection can potentially simplify the procedure and shorten the operative time. However, the residual sac tissue may increase the risk of postoperative seroma formation. Several studies have investigated Postoperative outcomes, with controversial results [8]. Therefore, we conducted a systematic review and meta-analysis to present a higher level of evidence concerning the management of the hernia sac in laparoscopic hernia repair using TEP or TAPP.

This systematic review and meta-analysis aimed to compare the postoperative outcomes of sac reduction and sac transection during laparoscopic mesh hernia repair.

## Methods

We conducted a meta-analysis according to the Preferred Reporting Items for Systematic Review and Meta-analysis (PRISMA) 2020 [9] and the AMSTAR 2 (Assessing the Methodological Quality of Systematic Reviews) guidelines [10]. The study protocol was registered in PROSPERO under the number ID: CRD42023391730.

### Electronic database searches

An extensive electronic search of relevant literature until December 10, 2022, with no language restrictions, was performed using the following databases: Cochrane Library’s Controlled Trials Registry and Database of Systematic Reviews, PubMed/MEDLINE of the United States National Library of Medicine, Google Scholar, Excerpta Medica Database (Embase), and Scopus. The keywords used were “Randomized Controlled Trials,” “Clinical Controlled Trials,” “inguinal hernia,” “hernia repair,” “hernioplasty,” “herniorrhaphy,” “laparoscopic hernioplasty,” “reduction,” “transection,” “dissection,” and “ligation” “total extraperitoneal,” “transabdominal preperitoneal,” “seroma,” and “morbidity.” We manually checked the reference lists of articles obtained for eligible clinical trials.

### Eligibility criteria

#### Studies

All randomized and controlled clinical trials reported comparisons between sac transaction and sac resection during laparoscopic mesh hernia repair. Non-comparative studies, editorials, letters to editors, review articles, and case series or papers were not considered in this study. We excluded clinical trials that compared sac transection and sac resection during open surgery or treatment without mesh repair.

#### Populations

Adults (aged ≥ 18 years) of either sex undergoing laparoscopic groin hernia repair using a mesh were included.

#### Intervention

Laparoscopic hernia repair with transection of the hernia sac (TS group).

#### Comparator

Laparoscopic hernia repair with resection of the hernia sac (RS group).

#### Outcomes measures

The main outcome measure was seroma formation. A seroma was defined in the original studies as a collection of fluid or swelling at the surgical site or in the scrotum. The secondary outcomes were morbidity, bleeding, operative time, postoperative pain, bleeding, reoperation, hospital stay, and recurrence.

### Data collection and analysis

#### Study selection

After independent literature research by two authors. The two authors independently reviewed all the abstracts. RCTs and CCTs were considered. The full texts of all the studies that met the inclusion criteria were retrieved. After consulting a third review team member, the discussion resolved any disagreements.

#### Assessment of studies quality and risk of bias assessment

Two authors independently appraised all studies that met the selection criteria. Concerning quality assessment, CCTs and RCTs were assessed according to the methodological index of non-randomized studies (MINORS) [11] and the Consolidated Standards of Reporting Trials (CONSORT) statement [12], respectively. We excluded all studies with a MINORS or CONSORT statement inferior to 13. For the risk of bias in the RCTs, we used the Cochrane tool for bias assessment to assess the risk of bias in randomized trials (RoB2) [13]. For the risk of bias in CCTs, we used the Newcastle Ottawa Scale (NOS) [14].

#### Data extraction

Two authors independently extracted data from the retained studies. Disparities were settled after a discussion with a third author. If studies presented the results as the median and interquartile range (IQR) or range, we converted the values to mean and SD according to Cochrane Handbook 7.7.3.5 [15] or Hozo et al. [16]., as appropriate.

#### Evaluation of effect size

We used the RevMan 5.4 statistical package from the Cochrane Collaboration for meta-analysis [17]. We selected the mean difference (MD) as an effective measure of continuous data. Odds ratios (OR) with 95% confidence intervals (95% CI) were calculated for dichotomous variables. A random effects model was used. The threshold of significance was set at p < 0.05.

#### Assessment of heterogeneity

We used the Cochrane Chi² test (Q-test), I² statistic, and variance TAU² to estimate the degree of heterogeneity [18]. Funnel plots were used to identify studies responsible for heterogeneity. A subgroup analysis was performed when all the included studies reported outcomes.

#### Summary of findings

Two authors independently assessed the evidence of the primary outcomes. We used The Grading of Recommendations Assessment, Development, and Evaluation (GRADE) [19]. We considered the study limitations in terms of the constancy of effect, imprecision, indirectness, and publication bias. We assessed the certainty of the evidence as high, moderate, low, or very low. We used GRADEpro GDT software to prepare a summary of the findings tables.

## Results

### Literature search results

**Fig. 1:**
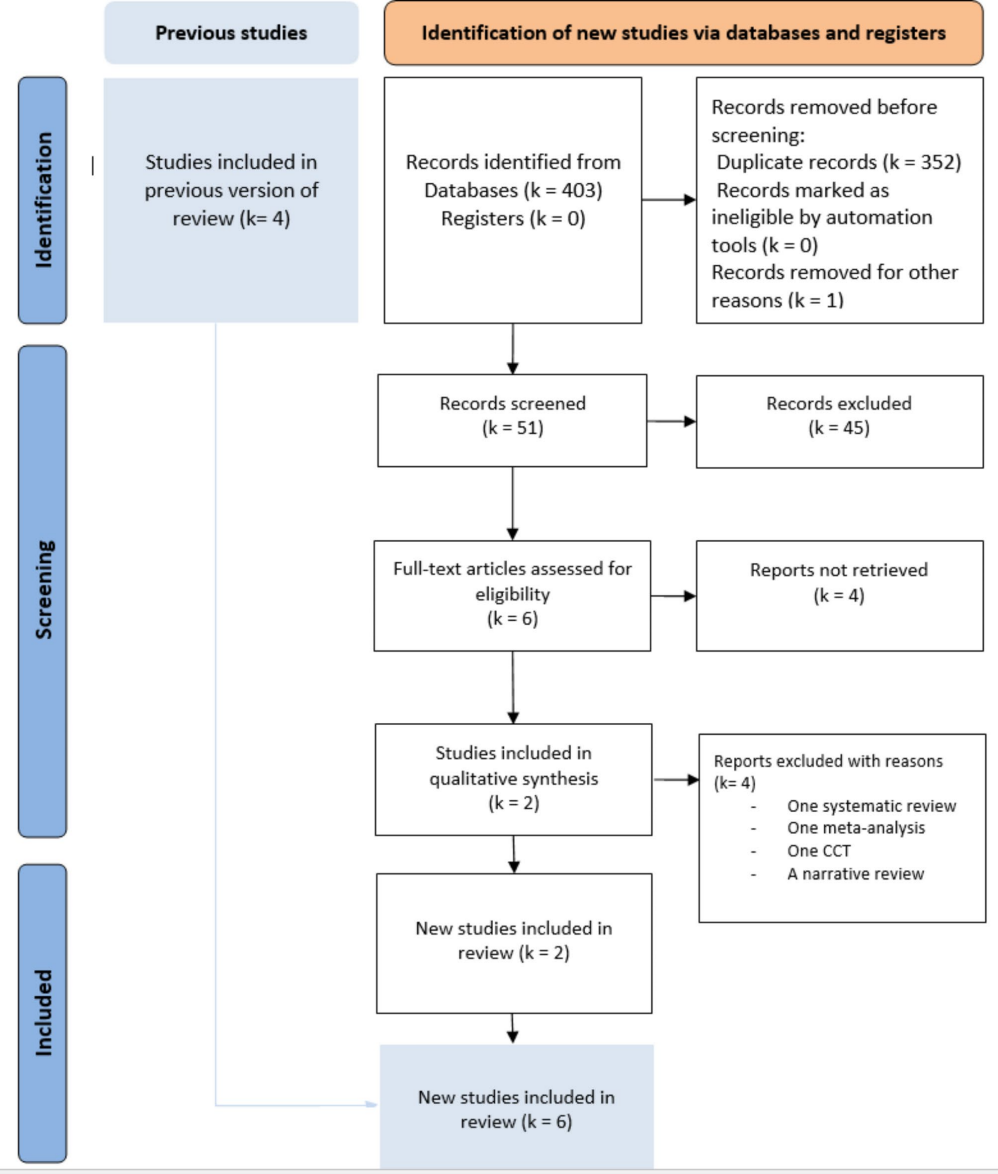
PRISMA 2020 flow diagram of the included studies

The literature search yielded six eligible studies [6, 20–24] (Fig. 1). Four studies were included in the previous version of the review [6, 20, 21, 24]. Two studies were RCTs [6, 24] and four studies were CCTs [20–23]. Four studies were excluded: one systematic review with a meta-analysis of this subject [8], one systematic review [25], one narrative review [26], and one CCT comparing hernia remnant sac fixation with no fixation [27]. The number of involved patients was 2941 patients:821 patients in the TS group and 2120 patients in the RS group, respectively. The list of the retained studies, NOS and RoB 2 scores of the included patients were presented in Table 1. The demographic data of the retained studies were presented in Table 2. Several studies have been published between 2002 and 2022. Five studies were conducted in China, and one study was conducted in Korea. The mean age of the patients ranged from 49 to 64 years. The sex ratio was six, with a large male predominance. Regarding laparoscopic hernia repair, three studies performed TEP hernia repair, two studies performed TAPP hernia repair, and one study performed TEP and TAPP. The follow-up ranged from one to 40.8 months.

**Table 1:** List of the included studies, quality assessment, and the risk of bias evaluation

First author	Journal	Year of publication	Study design	Country	Study period	Quality assessment (MINORS/CONSORT)	New-Castle Ottawa scale			Cochrane risk of bias 2					
							Selection	Comparability	Outcome	Randomization process	Deviations from intended interventions	Bias in measurement of outcome	Bias to missing outcome data	Bias in selecting the reported results	Overall bias
Choi et al.	Journal of laparoendoscopic & advanced surgical techniques	2011	CCT	Korea	July 2003 - December 2008	20	**	**	**	-	-	-	-	-	-
Lau et al.	Journal of laparoendoscopic & advanced surgical techniques	2002	CCT	China	September 1999 - July 2001	20	**	**	**	-	-	-	-	-	-
Lei-liu et al.	Asian Journal of Surgery	2022	CCT	China	January 2020 - June 2021	19	***	**	**	-	-	-	-	-	-
Li et al.	Surgical Endoscopy	2019	RCT	China	May 2017 - May 2018,	19	-	-	-	Low risk	Low risk	Low risk	Low risk	Low risk	Low risk
Pan et al.	*BMC Surgery*	2022	CCT	China	January 2017 - January 2019	18	***	**	**	-	-	-	-	-	-
Ruze et al.	Surgical Endoscopy	2018	RCT	China	May 2015 - September 2017	20	-	-	-	Low risk	Some concerns	Low risk	Low risk	Low risk	Some concerns

MINORS: Methodological index for non-randomized studies; CONSORT: Consolidated Standards of Reporting Trails; CCT: clinical controlled trials; Randomized controlled trials

**Table 2:** Characteristics of the retained studies in the meta-analysis

First author	Age (year)	Sex (M/F)	Total number	TS group	RS group	Surgical technique	Hernia type (unilateral/bilateral)		Hernia side (right/left)		Follow-up (month)
							TS group	RS group	TS group	RS group	
Choi et al.	49	476/44	520	275	245	TEP	-	-	100/99	175/146	40.8
Lau et al.	64	96/3	99	34	65	TEP	-	-	-	-	6
Lei-liu et al.	57.37	330/0	330	90	240	TAPP	88/213	10/27	-	-	1
Li et al.	61.9	70/0	70	35	35	TEP	31/30	4/5	-	-	12.4
Pan et al.	≥ 60:541; <60:1215	1613/180	1763	311	1452	TAPP/TEP	-	-	145/524	221/873	6
Ruze et al.	51.75	159/0	159	76	83	TAPP	71/63	12/13	-	-	3.1

M: male; F: female; TS: sac transection; RS: sac reduction; TEP: totally extraperitoneal hernia repair; TAPP: trans-abdominal preperitoneal hernia repair

### Outcome measures

#### Seroma

**Fig. 2:**
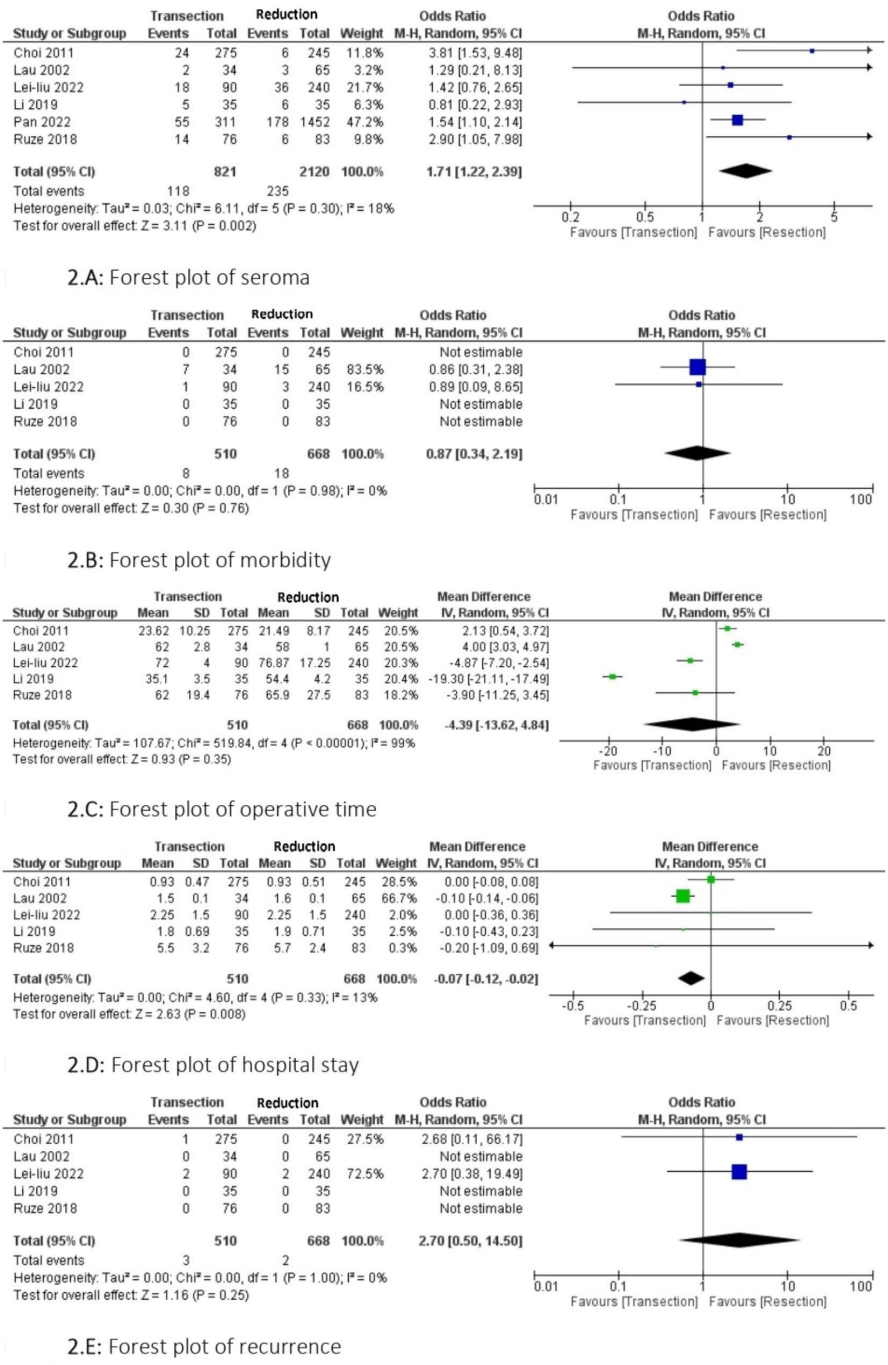
Forest plot of the different outcomes

All retained studies assessed seroma [6, 20–24]. I was reported in 118 of the 821 patients in the TS group and 235 of the 2120 patients in the RS group. There was a significantly lower seroma rate in the RS group than that in the TS group (OR = 1.71; 95% CI [1.22, 2.39], p = 0.002). There was low heterogeneity among the studies **(**Fig. 2.**A)**.

#### Morbidity

The morbidity rate was assessed in five studies [20–22, 24]. It was reported in eight of 510 patients in the TS group and 18 of 668 patients in the RS group. The difference between the two groups in terms of morbidity was not statistically significant (OR = 0.87; 95% CI [0.34, 2.19], p = 0.76). No heterogeneity was observed among the studies **(**Fig. 2.**B)**.

#### Operative time

The operative time was reported in five studies [20–22, 24]. It was assessed in 510 and 668 patients in the TS and RS groups, respectively. There was no statistically significant difference between the two groups in terms of operative time (MD = -4.39; 95% CI [-13.62, 4.84], p = 0.35). There was high heterogeneity among the studies Tau2 = 107.67 (I²=99%) **(**Fig. 2.**C)**.

#### Hospital stay

Hospital stay was reported in five studies [20–22, 24]. It was assessed in 510 and 668 patients in the TS and RS groups, respectively. There was a significantly shorter hospital stay in the TS group (MD = -0.07; 95% CI [-0.12, -0.02], p = 0.008). No heterogeneity was observed among the studies **(**Fig. 2.**D)**.

#### Recurrence

The recurrence rate was assessed in five studies [20–22, 24]. It was reported in three of 510 patients in the TS group and two in 668 patients in the RS group. The difference in recurrence between the two groups was not statistically significant (OR = 2.70; 95% CI [0.50, 14.50], p = 0.25). No heterogeneity was observed among the studies **(**Fig. 2.**E)**.

#### Postoperative pain

Postoperative pain was assessed in four studies [20, 21, 24, 28]. Different measurement features were used in these studies, and a pooled analysis was not feasible for performing a meta-analysis. All the studies concluded that there was no difference between the two groups. The results are summarized in Table 3.

**Table 3:** Postoperative pain according to the different studies

Studies	Methods	Results
Choi 2011	Patients who needed more than two doses of analgesics for operative site pain were recorded.	Postoperative pain was occurred in seven patients in the TS group and six patients in the RS group (p = 0.994).
Lau 2002	The severity of pain at rest and on coughing was assessed daily with a linear analogue pain score on a scale from0 to 10 after the operation. During the hospital stay, the surgeon determined the pain score during the ward round. After discharge, all patients were taught to fill in a pain score chart at home daily to document pain at rest and on coughing. All pain score charts were collected by the surgeon during the first follow-up clinic visit.	No difference in postoperative daily pain scores at rest and on coughing between the two groups (p = NS).
Li 2019	Pain was measured by visual analog scale (VAS) (range: 0–10), a 10 cm line was drawn and marked equidistant 1–10, with 0 representing no pain and 10 representing the most severe pain. Patients with VAS greater than 5 were considered to have acute pain or chronic pain.	There were no significant differences between the two groups in the level of acute pain (p = 0.73). During the follow-up period, there were no chronic pain issues observed in either group.
Ruze 2018	Postoperative pain based on a visual analog scale where 0 indicated no pain and 10 indicated the worst pain imaginable. Pain was determined at seven days, one month, and three months.	Pain at seven days p = 0.502 Pain at one-month p = 0.933 Pain at three months p = 0.285

### Reporting of the effects of transection of hernia sac during laparoscopic hernia repair

A Summary of the evidence is presented in Table 4. This review shows that when the hernia sac is transected:

- This may have reduced the seroma rate with a shorter hospital stay.
- We do not know if it leads to additional morbidity, recurrence, operative time, or postoperative pain because the evidence regarding these outcomes is very uncertain.

**Table 4:** Summary of findings table

Outcomes	№ of participants (studies) Follow-up	Certainty of the evidence (GRADE)	Relative effect (95% CI)	Anticipated absolute effects	
				Risk with reduction	Risk difference with transection
Seroma	2941 (2 RCTs + 4 CCTs)	⨁⨁◯◯ Low^a^	OR 1.71 (1.22 to 2.39)	111 per 1 000	65 more per 1 000 (21 more to 119 more)
Morbidity	1178 (2 RCTs + 3 CCTs)	⨁◯◯◯ Very low^a^	OR 0.87 (0.34 to 2.19)	Low	
				0 per 1 000	0 fewer per 1 000 (0 fewer to 0 fewer)
Recurrence	1178 (2 RCTs + 3 CCTs)	⨁◯◯◯ Very low^a^	OR 2.7 (0.5 to 14.5)	3 per 1 000	5 more per 1 000 (1 fewer to 39 more)
Operative time	1178 (2 RCTs + 3 CCTs)	⨁◯◯◯ Very low^a,b^	-	-	MD 4.39 Min lower (13.62 lower to 4.84 higher)
Hospital stay	1178 (2 RCTs + 3 CCTs)	⨁◯◯◯ Very low^a,b^	-	-	MD 0.07 min lower (0.12 lower to 0.02 lower)

*The risk in the intervention group (and its 95% confidence interval) is based on the assumed risk in the comparison group and the relative effect of the intervention (and its 95% CI). CI: confidence interval; MD: mean difference; OR: odds ratio

GRADE Working Group grades of evidence

High certainty: we are very confident that the true effect lies close to that of the estimate of the effect.

Moderate certainty: we are moderately confident in the effect estimate: the true effect is likely to be close to the estimate of the effect, but there is a possibility that it is substantially different.

Low certainty: our confidence in the effect estimate is limited: the true effect may be substantially different from the estimate of the effect.

Very low certainty: we have very little confidence in the effect estimate: the true effect is likely to be substantially different from the estimate of effect.

*Explanations*

a. Small sample size of patient, inferior to 400 patients

b. Heterogeneity among the retained studies

## Discussion

This systematic review and meta-analysis showed that hernia sac transection is associated with a lower seroma rate and shorter hospital stay with similar morbidity, operative time, recurrence, and postoperative pain compared to the reduction of the hernia sac.

The proper management of the hernia sac during laparoscopic repair is crucial, and cutting it could simplify the procedure by eliminating the challenging dissection of the sac from the spermatic cord. Our research found both techniques to be safe and feasible, with comparable rates of complications. Nevertheless, previous studies have identified a higher incidence of postoperative seroma [8, 24]. This complication was defined as fluid exudation and accumulation in the surgical field. It is the most common postoperative complication after laparoscopic inguinal hernia repair, with various reported rates ranging from 1.9 to 11.7% [21]. The variation in reported incidence rates of seroma among studies can be attributed to the fact that most cases of seroma are asymptomatic and resolve spontaneously without treatment. Susmallian et al. [29] suggested that seroma was diagnosed clinically in only 35% of cases, whereas ultrasound examination revealed the presence of seroma in 100% of patients, and the amount of fluid collection increased until the 7th day after surgery and decreased after laparoscopic repair of the incisional hernia. Morales-Conde et al. [30] created a seroma classification system in which they defined seroma as a complication only if they were symptomatic, persisted for longer than six months, or were infected (types III and IV). Clinically relevant seroma that disappeared in less than six months (types I and II) were classified as incidental findings, reflecting that these were considered normal sequelae of the operation. Type III seroma persists for longer than six months or becomes symptomatic but does not require intervention, while type IV seroma is symptomatic and requires intervention. In this classification, only seroma types III and IV should be considered as real complications as they affect the clinical progression of the patient. Several reasons have been attributed to the development of seroma formation after laparoscopic inguinal hernia repair, including dissection of the preperitoneal space for mesh placement, the existence of dead space after hernia sac reduction, and irrigation of prosthetic materials implanted in the preperitoneal space [31]. According to our study, the management of the distal sac, reduction, or transection of the hernia sac in inguinal hernia repair affects the occurrence of postoperative seroma. This is in agreement with the International Endohernia Society guidelines published in 2015 [32], which reported that the complete reduction of the hernia sac may eliminate the occurrence of chronic seroma or pseudo-hydrocele. In addition, in a recent systematic review of the literature, Li et al. [25] reviewed of literature how enrolled four studies that compared the results of indirect hernia sac transection and complete sac reduction. The pooled results indicated that indirect hernia sac transection was associated with an increased seroma rate. A meta-analysis performed by Chai et al. [8], which included 848 patients, concluded that sac transection may increase the risk of seroma formation. Several therapeutic modalities have been reported to prevent seroma formation. We thought that even the heterogeneity among the different included studies in our review was due to a non-standardized diagnostic criterion of Postoperative seroma or if they had used any surgical features to reduce the seroma rate. A systemic review published by Li et al. [28] mentioned six adjunctive techniques to reduce seroma formation: transversalis fascia inversion with tacking, the endoloop technique, barbed suture closure of the transversalis fascia, surgical drains, and fibrin sealant. This systematic review concluded that seroma formation is a natural process that cannot be completely prevented following laparoscopic inguinal hernioplasty, particularly in patients with direct and large indirect inguinal hernias. Some intraoperative adjunctive techniques are effective in reducing clinically palpable seroma formation in selected patients. The way a hernia sac is managed during laparoscopic inguinal hernia surgery can impact the duration of hospitalization. Hospital stay duration is commonly used as an indicator of efficiency, and there have been numerous studies investigating this topic with conflicting outcomes. In a systematic review of the literature, Li et al. [25] found no statistically significant difference in the length of hospital stay between the two procedures. However, these findings were consistent with those of Chai et al. [8], who reported a significantly shorter hospital stay after sac transection than after sac reduction. Although advances in surgical techniques and the use of meshes have improved outcomes for inguinal hernia repair, recurrence rates remain a significant concern, ranging from 1 to 7.9% [33, 34]. Recurrence of inguinal hernia is a possibility at any point following surgery. Various risk factors, both modifiable and non-modifiable, are responsible for its occurrence, such as factors related to the patient and surgical techniques. SiddaiahSubramanya et al. [35] concluded that higher BMI, smoking, diabetes, and postoperative surgical site infections increase the risk of recurrence and can be modified accordingly. In addition to surgical techniques such as using a larger mesh with better tissue overlap, reducing recurrence rates after inguinal hernia surgery can also depend on the surgeon’s experience. The way the hernia sac is managed during surgery can also have an impact on recurrence rates. The Swedish Hernia Register found that the 5-year cumulative incidence of reoperation for recurrence after open inguinal hernia repair was 1.7% for hernia sac excision, 1.7% for division, and 2.7% for invagination. For indirect hernia repair, sac excision and sac division were associated with a lower relative risk of reoperation for recurrence compared to sac invagination. Lichtenstein repair with hernia sac excision had a 5-year cumulative reoperation incidence for a recurrence rate of only 1%. The authors concluded that excision of the indirect hernia sac in inguinal hernia repair is associated with a lower risk of hernia recurrence than division or invagination [36]. Chai et al. [8] concluded that there was no difference in terms of recurrence between the sac transaction and sac reduction groups, which is similar to our findings. Regarding postoperative pain, Othman et al. [37] compared the effect of invagination excision of the hernia sac without ligation with the traditional method of high ligation of the hernia sac on postoperative pain and recurrence. The authors found that invagination and excision of the hernia sac were safe and suitable for repairing sliding hernias without any adverse effects. They recommend against ligating the hernia sac in inguinal hernia surgery, as it is unnecessary, time-consuming, and associated with increased postoperative pain. Choi et al. [20] recorded the frequency at which patients required more than two analgesic doses. Lau et al. [21] evaluated pain scores at rest and on daily coughing for the first four postoperative days. Li et al. [28] defined a visual analogue scale pain score > 5 as significant pain. Ruze et al. [24] assessed pain scores on the seventh postoperative day, at one and three months following surgery. No significant differences were observed between the two groups.

Compared with the previous version of this systematic review and meta-analysis published by Choi et al. [20], we have included two additional recent studies with the highest number of patients: 2941 patients versus 848 patients. We have assessed additional outcomes like hospital stay and recurrence. In addition in our study, we have found a significant difference between the two groups in terms of hospital stay which is novel and interesting. Furthermore, we have used the most updated methodology of systematic review and meta-analysis and we have performed a GRADE assessment for suitable conclusions. On the other side, this study presented several limitations. Owing to the small number of RCTs, lack of some outcomes, and lack of long-term follow-up, we included additional CCTs. The quality of the included studies was limited by their retrospective nature, and the certainty of the evidence was very low for some outcomes. Therefore, further prospective and larger studies are required to confirm these findings. We did not assess other outcomes such as postoperative pain, time of return to activities, chronic pain, and long-term discomfort. The risk of bias assessment using NOS and Cochrane RoB-2 was performed, and there was no high risk of bias in the retained studies. It is interesting to note that five out of six studies came from China and only one from Korea. None from European countries, Africa, or the United States. Therefore, we cannot speculate on the generalizability of the results of hernia sac resection or reduction in laparoscopic hernia repair. The number of included patients in our study was 2941 patients. A larger number of patients included was in 1763 patients by Pan et al. [23], which allowed us to reach statistical significance in some parameters. The systematic review and meta-analysis by Chai et al. [8] included only four studies. In addition, there was no summary of the findings table, and the study was not conducted according to the PRISMA guidelines 2020.

In conclusion, our study showed that hernia sac transection is associated with lower seroma and shorter hospital stay with similar morbidity, operative time, recurrence, and postoperative pain compared to the reduction of the hernia sac. For better placement of the best modality for hernia sac management during laparoscopic hernia repair, additional multicenter RCTs with larger sample sizes are required.

## Data Availability

All data generated or analyzed during this study are included in this published article.
